# Outcome from out-of-hospital cardiac arrest managed by the pre-hospital emergency medical system in Martinique, a French Caribbean Overseas Territory

**DOI:** 10.1016/j.resplu.2024.100847

**Published:** 2024-12-18

**Authors:** Florian Negrello, Jonathan Florentin, Romain Jouffroy, Vianney Aquilina, Rishika Banydeen, Rémi Neviere, Dabor Resiere, Moustapha Drame, Papa Gueye

**Affiliations:** a Department of Emergency Medicine, SAMU 972, University Hospital of Martinique (CHU de Martinique), Fort-de-France, Martinique, France; b Cardiovascular Research Team (UR5_3 PC2E), University of the French West Indies (Université des Antilles), Fort de France, Martinique, France; c Department of Emergency Medicine, University Hospital of Martinique (CHU de Martinique), Fort-de-France, Martinique, France; d Intensive Care Unit, Ambroise Paré Hospital, Assistance Publique - Hôpitaux de Paris, Boulogne Billancourt, France; e Research Center in Epidemiology and Population Health - U1018 INSERM, Saclay, Paris Saclay University, France; f Institute of Biomedical Research and Sports Epidemiology - EA7329, INSEP, Paris, Paris University, France; g Department of Critical Care Medicine, Emergency Medicine and Toxicology, University Hospital of Martinique (CHU de Martinique), Fort-de-France, Martinique, France; h Department of Cardiology, University Hospital of Martinique (CHU de Martinique), Fort de France, Martinique, France; i EpiCliV Research Unit, University of the French West Indies (Université des Antilles), Fort-de-France, Martinique, France; j Department of Clinical Research and Innovation, University Hospital of Martinique (CHU de Martinique), Fort de France, Martinique, France

**Keywords:** Out-of-hospital cardiac arrest, Cardiopulmonary resuscitation, Outcomes, Survival, Cerebral Performance Category

## Abstract

**Introduction:**

Out-of-hospital cardiac arrest (OHCA) affects approximately 46,000 people in France annually and survival remains low. There is no published data specific to the characteristics and outcomes of OHCA in French overseas territories, especially in the French Caribbean territories. The aim of this study was to describe the characteristics and outcomes of adult OHCA patients managed by the Emergency Medical Service team (EMS) in Martinique.

**Methods:**

All adults with OHCA, managed by the EMS of Martinique between January 1st 2018 and June 30th 2019, were included. Primary outcome was 30 day-survival and neurological outcome at 30 days assessed by the Cerebral Performance Category scale (CPC). Secondary outcomes were return of spontaneous circulation (ROSC) prior to hospital admission and causes of cardiac arrest in patients with ROSC.

**Results:**

This study included 340 OHCA patients. The population was predominantly male (64%), with a median age of 68 [54–78] years. OHCA resulted from a medical condition in 314 patients (92%) and occurred mainly at home (75%), in the presence of witnesses for 235 patients (69%). Basic life support was initiated in 174 OHCA (51%). Median time to first-responders’ and prehospital mobile intensive care unit’s arrivals at scene were 17 [10–30] and 27 [19–41] minutes after call to the EMS dispatching center for OHCA. Non-shockable initial rhythm was present in 315 patients (93%), and 240 patients (71%) received advanced life support. Thirty-one patients (9%) achieved ROSC. On day 30, 13 patients (3.8%) were still alive, and 8 of them (2.4%) were alive with a CPC score of 1 or 2.

**Conclusion:**

The overall adult OHCA survival rate and survival with good neurological status on day-30 in the French Caribbean island of Martinique are low. OHCA survival rate may be improved by educating the population on basic life support techniques and reducing the time responses for first-responders and prehospital mobile intensive care unit to reach patients.

## Introduction

Out-of-hospital cardiac arrest (OHCA) affects annually approximately 89 per 100,000 adults in the United States^1^ and 62 per 100,000 inhabitants in France. Despite all the technological and medical advances of the 21st century, OHCA survival rate remains low (below 10%) worldwide.^2^

Cardiac arrest (CA) chain of survival is the major point to improve OHCA outcomes, especially if this chain is set up quickly.^3^ Early recognition and early call to the emergency medical service (EMS), witness basic life support (BLS), defibrillator public access and use, quick arrival of the emergency medical system team for cardiopulmonary resuscitation (CPR), and advanced life support (ALS) are the described links in the CA survival chain.^4^ Access to public defibrillators with early defibrillation, when necessary, are consistently associated with an overall shockable OHCA survival improvement.^5^, ^6^ Survival and functionally favorable outcomes are better when a bystander, rather than the EMS team, provides the initial shock^7^ and initiates CPR.^8^, ^9^

To the best of our knowledge, there is no published data concerning the outcome of OHCA in the French overseas territories, especially in the French Caribbean territories. The aim of this study was to describe the characteristics and outcomes of the largest cohort of adult OHCA patients managed by the EMS team in Martinique, one of the French Caribbean Overseas territories. These knowledges are important for highlighting local differences to improve care.

## Methods

### Study design and setting

We retrospectively analyzed data recorded in the electronic cardiac arrest registry “ReAC”.^10^, ^11^ ReAC is a French national registry, also including data from the French Caribbean island of Martinique. With 360,749 inhabitants registered in 2017 on 1,130 km^2^ (436squaremiles), Martinique is a densely populated island (319inhabitantsperkm2).^12^

The Martinique EMS is a dual system, where first-responders (exclusively firefighters) provide basic life support (BLS), while a prehospital mobile intensive care unit (MICU) provides advanced life support (ALS) in the field. The MICU team includes at least one ambulance driver, one nurse, and one trained emergency physician.^13^ The means available for the management of cardiac arrests by the MICU include all the treatments and medical devices indicated in the management of cardiac arrests, except extracorporeal cardiopulmonary resuscitation. A call reception and dispatching center with a single number 15 is part of the EMS based at the University Hospital of Martinique and named SAMU 972. Upon receiving a call from a relative or witness, the dispatcher physician simultaneously sends firefighters and the MICU team to the scene. Most of the time, firefighters arrive earlier at the scene allowing an earlier BLS initiation. A detailed description of the French EMS has been previously published.^14^ Martinique hospital resources correspond to the French standards for a University Hospital center with an EMS, an Emergency Department (ED), an intensive care unit (ICU), a cardiac ICU, a 24-hour percutaneous coronary intervention unit, a circulatory support unit for extracorporeal cardiopulmonary resuscitation, 24-hour radiology department, 24-hour biology department, functional explorations with electroencephalogram and a rehabilitation unit.

### Data collection

We analyzed all OHCA treated managed by EMS with MICU intervention between January 1st 2018 and June 30th 2019. Inclusion criteria were: patients aged 18 years and above with complete registry records. Non-inclusion criteria were end-of-life, expected death, confirmed death without resuscitation attempt, corpse discoveries and in-hospital cardiac arrest.

In order to ensure exhaustive identification of OHCA cases during the study period, patient records were cross-referenced between the call reception and dispatching center database (*Centaure 15.V4™*) and the ReAC registry. The search terms used for patient identification on Centaure 15.V4™ database were: “cardiac arrest”, “sudden death” and “death” with MICU intervention. The ReAC registry was continuously updated with new patients that were identified on Centaure 15.V4™. Anonymized data extraction was conducted on October 30th 2020.

All data, including Utstein characteristics of OHCA were recorded.^15^, ^16^ Utstein characteristics included sex, age, medical history, location, presence of witnesses, bystander CPR initiation, resuscitation time intervals from cardiac arrest time, initial cardiac rhythm, extra corporeal life support use, angioplasty intervention, presumed cause of cardiac arrest, presence/absence of return of spontaneous circulation (ROSC), and presence/absence of hospital admission. Vital status at hospital discharge and at 30 days, and neurological outcome at 30 days were collected.

### Outcomes

The primary outcome was 30 day-survival and neurological outcome at 30 days assessed by the Cerebral Performance Category (CPC) scale. CPC is a five-point scale used to categorize neurological outcome after cardiac arrest,^17^ with categories 1 and 2 considered to be a good outcome, categories 3 and 4 considered a poor outcome, and category 5 signifying death. Secondary outcomes were: ROSC prior to hospital admission and causes of cardiac arrest in patients with ROSC.

### Ethical approval

This study was conducted in accordance with the Declaration of Helsinki and French laws related to biomedical research. The national registry ReAC had an authorization of the French National Data Protection Commission (CNIL authorization no. 910,946 on 17/08/2015) for the use of patient data for research purposes. This study was further approved by the Institutional Review Board of the University Hospital of Martinique on October 20th 2020 (IRB no. 2020/080).

### Statistical analysis

For quantitative variables, median with interquartile ranges (IQR) were calculated. Categorial variables were presented as a number of events and percentages. Statistical comparisons were not performed because of the low number of patient outcomes.

## Results

**Fig. 1:**
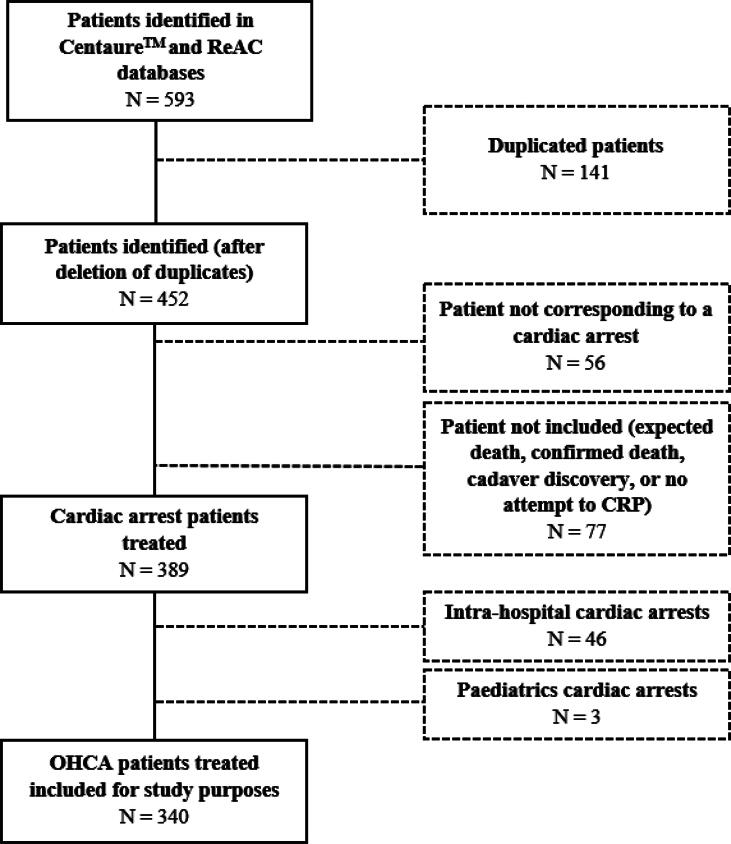
Flow chart of Out-of-Hospital Cardiac Arrest (OHCA) patients between January 1st 2018 and June 30th 2019, in Martinique. *Abbreviations: OHCA: CPR: Cardio-Pulmonary resuscitation; Out-of-hospital cardiac arrest; ReAC: Registre électronique des arrêts cardiaques (Electronic Cardiac Arrest Registry)*.

During the 18-month study period, 593 OHCA were identified, among which 253 patients were ineligible (Fig. 1). As such, 340 adult OHCA patients were finally included in the study analysis (Fig. 1), corresponding to an overall estimated annual incidence of 61.4 per 100,000 inhabitants during the study period.

Sample characteristics are summarized in Table 1. The sample was predominantly male (64%), with a median age of 68 [54–78] years. OHCA was caused by a medical condition in 314 patients (92%) and occurred mainly at home for 254 patients (75%), with a witness presence in 69% of cases. Non-shockable initial rhythm was present in 315 patients (93%), and 240 patients (71%) had ALS by MICU team. Thirty-three cardiac arrests (10%) were not identified by the EMS call center at the time of the call, and were finally diagnosed by the first-responders on scene arrival. Thirty-six patients (11%) were transported to the University Hospital of Martinique. Overall, 31 patients (9%) presented ROSC, and 5 patients (2%) with refractory cardiac arrest were transported with automated CPR device for the purpose of extracorporeal cardiopulmonary resuscitation.

**Table 1:** Out-of-hospital cardiac arrest population characteristics between January 1st 2018 and June 30th 2019, in Martinique.

Population characteristics (n = 340)	Statistical parameters
Age, years	68 [54–78]
Age group, years	
18–65	144 (42.4)
65–90	181 (53.2)
> 90	13 (3.8)
Sex	
Women	122 (35.9)
Men	218 (64.1)
Place of intervention	
Home / Private place	254 (74.7)
Public road or public place	68 (20.0)
Other	18 (5.3)
Witness of OHCA	
Bystander witnessed	235 (69.1)
EMS witnessed	32 (9.4)
Unwitnessed	73 (21.5)
Type of OHCA	
Medical	312 (91.8)
Traumatic	28 (8.2)
Supposed cause of OHCA	
Cardiac	235 (69.1)
Respiratory	40 (11.8)
Drowning	9 (2.6)
Neurologic	18 (5.3)
Public road accident	10 (2.9)
Firearm / Stab wound	10 (2.9)
Hanging	4 (1.2)
Other	10 (2.9)
BLS before MICU arrival	
Bystander CPR	174 (51.2)
Public access AED	11 (3.2)
First-responders AED	239 (70.3)
Electric shock delivered (public AED)	2 (0.6)
Electric shock delivered (FR)	27 (7.9)
Durations (min) *	
Time to first compression	8 [1–17]
First AED analysis	18 [11–27]
First-responder response time	17 [10–30]
MICU response time	27 [19–41]
Initial rhythm with MICU	
Asystole	296 (87.1)
Electric activity without pulse	19 (5.6)
Ventricular fibrillation or tachycardia	18 (5.3)
Return of spontaneous circulation	6 (1.8)
Advanced life support by MICU	
Yes	240 (70.6)
No	93 (27.4)
Not applicable (ROSC)	6 (1.8)

Abbreviations: OHCA: Out-of-hospital cardiac arrest; FR: First-responders; CPR: CardioPulmonary Resuscitation; BLS: Basic life support; AED: Automatized external defibrillator; MICU: mobile intensive care unit ROSC: Return of spontaneous circulation. Data are expressed as median and interquartile range for quantitative variables, or as absolute number and percentages for qualitative variables. *Duration time is calculated between estimated time of cardiac arrest or time of call and first thoracic compression, first AED analysis, first responder arrival, and MICU arrival.

Twenty-six (10.6%) patients were admitted alive to the hospital, 31 with a ROSC and five with automated CPR device. The duration between OHCA occurrence and hospital admission was 103 ± 48 min, 108 ± 48 min for patients with ROSC and 74 ± 37 min for patients in the process to resuscitation. Two patients with refractory cardiac arrest were implanted by E-CPR. The admission unit was mainly emergency room (20patients), then intensive care unit (8patients) and coronary angiography unit (8patients). OHCA causes were cardiac for 21 patients, 3 patients with acute respiratory failure, 5 patients with acute neurologic lesion, 2 drownings, 1 poisoning and 4 other causes (septic shock and metabolic disorders). On day 30, 13 patients (3.8%) were still alive, and 8 of them (2.4%) were alive with a CPC score of 1 or 2.

Results from the cohort of OHCA patients managed by the EMS team in Martinique contrast with previous reports of OHCA patients from the national French registry, ReAC (supplement table).

## Discussion

To the best of our knowledge, this is the first descriptive study of adult OHCA patients’ outcome in a French Caribbean territory. The observed survival rate in Martinique is lower than the national average in mainland France and Europe.^2^, ^18^, ^19^, ^20^ Several reasons could explain this low OHCA survival rate in Martinique. Firstly, delays for on-scene arrival of first-responders are very long, occurring on average 20 min, contrary to French or international published data which report delays between 3 and 10 minutes.^19^, ^21^, ^22^, ^23^ Secondly, MICU team response times are also longer than the national French EMS times, which is around 18 to 20 minutes.^2^, ^24^, ^25^ This delay may be explained by geographical reasons: Martinique is an island with a mountainous and rural part, a sparse road network, places that require special consideration and are difficult to reach, such as beaches, islets, and hiking trails, and areas that are very far from the island center, where the University Hospital of Martinique’s EMS are located. Thirdly, the absence of a volunteer first responder-system could have a negative effect on the start of BLS, as well as patient outcome.^6^, ^9^, ^26^, ^27^, ^28^, ^29^ Return of spontaneous circulation (ROSC) and OHCA survival rates are therefore higher in regions that have a dispatched first responder system^30^, ^31^ and European experts in OHCA management recommend implementing this system in all EMS.^32^

Although around 70% of cases in our study sample were witnessed. The present study found that most OHCA occurred at home (75%), with bystander witnessed (69%). The observed presence of a witness at the moment of cardiac arrest in our study population is similar to or higher than what is reported the French cohorts^2^, ^10^, ^24^, ^33^ or European cohorts.^34^ The prompt identification of OHCA and immediate initiation of BLS are crucial to decrease the no-flow duration, as well as to increase survival and OHCA outcomes. The low rate of resuscitation by witness or relatives may result from several causes. Firstly, the absence of chest compression by witnesses is mainly due to the lack of training in BLS or a fear of performing first aid gestures despite potential telephone-guided resuscitation. Secondly, accurate identification of cardiac arrest is a challenge for witnesses or relatives, just as it is sometimes for the emergency call center.^4^, ^18^, ^21^ Indeed, 10% of cardiac arrests were not identified by the EMS call center at the time of the call, and were finally diagnosed by the first-responders. The reported ROSC rate (9.1%) in our study is half the survival rate observed in other French and European studies of 17.2% and 25.2% respectively.^10^, ^34^ In Northern European countries, a synergy of multiple actions and higher survival rates with good neurological outcome have been reported.^6^, ^27^, ^35^, ^36^, ^37^ The OHCA survival rate varies across countries, but also within the same country and the time period considered.^38^ According to a *meta*-analysis of studies from 1980 to 2008, the OHCA survival rate at hospital discharge was 7.6%.^4^ Survival rate at day-30 in 28 countries in Europe varied from 3.1% to 20.4%, with good neurological status ranging from 2.8% to 18.2%.^19^ Survival to hospital discharge was higher in patients when a bystander performed chest compressions with ventilation (14%), compared to chest compressions only (8%).^19^ In addition to the presence of bystanders and the start of BLS without any delay, the presence of a “shockable” rhythm during early heart rhythm analysis positively influenced outcomes.^35^ Factors that may also explain the differences in survival rates in the studied French Caribbean population, independently of OHCA management, are the high level of precariousness with twice the rate of people living below the poverty line than in France mainland with its health consequences.^39^

In our study, nearly 90% of OHCA patients experienced asystole as their first rhythm recorded by MICU, higher than that of the series published in France,^2^, ^10^, ^18^, ^24^, ^33^ probably due to longer arrival times of first-responders and MICU, associated with a worse prognosis.^21^, ^40^, ^41^, ^42^, ^43^ OHCA survival outcome is improved if the automated external defibrillator is used before arrival Y.^44^, ^45^ Public access defibrillation in Martinique is limited, as they were only used on 11 patients (3%) in the present study, while defibrillation was delivered in 2 cases.

There are some limitations to this study, particularly because of its retrospective and single center design. However, data completeness and reliability are good, as patient data were systematically recorded by the MICU immediately after their return from intervention sites. Data input to the emergency software was carried out via standardized electronic case report forms, further guaranteeing good data quality, accuracy and completeness. Despite the similarities in medical resources and care provision between Martinique and other French overseas territories, the present results cannot be extrapolated to all of them, because of country specificities.

## Conclusion

The overall OHCA survival rate and survival with good neurological status at day-30 in adults residing on the French Caribbean island of Martinique are lower than those observed in mainland France. Despite the presence of witnesses, only half of them began BLS before the delayed on scene arrival of the first-responders and prehospital MICU. These points may contribute to the lower survival rate. OHCA survival can be improved by educating the population on basic life support techniques and improving the time arrival of first-responders and emergency services, through the better location of patients and smoother travel conditions to reach patients. Further research is needed to demonstrate the impact of these actions.

## CRediT authorship contribution statement

**Florian Negrello:** Writing – original draft, Methodology, Investigation, Conceptualization. **Jonathan Florentin:** Writing – review & editing. **Romain Jouffroy:** Writing – original draft. **Vianney Aquilina:** Methodology, Conceptualization. **Rishika Banydeen:** Writing – review & editing. **Rémi Neviere:** Writing – review & editing. **Dabor Resiere:** Writing – review & editing. **Moustapha Drame:** Writing – review & editing, Writing – original draft. **Papa Gueye:** Writing – original draft, Supervision, Methodology, Conceptualization.

## Declaration of competing interest

The authors declare that they have no known competing financial interests or personal relationships that could have appeared to influence the work reported in this paper.
