# Supplementary material for: Outcome from out-of-hospital cardiac arrest managed by the pre-hospital emergency medical system in Martinique, a French Caribbean Overseas Territory
Source: Resusc Plus. 2024 Dec 18;21:100847. doi: 10.1016/j.resplu.2024.100847 (PMC11780975; doi:10.1016/j.resplu.2024.100847)
Supplement: Supplementary Data 1 [file mmc1.docx]

**Supplement table:** Comparing the characteristics and outcomes of OHCA patients in Martinique with those registered in the French REAC registry

| **Authors**  **Setting**  **Period** | **Age, years** | **Sex**  **(% of males)** | **Home or private place**  **/**  **Public place** | **OHCA with**  **bystander witness** | **Medical**  **/**  **Traumatic** | **Bystander CPR** | **Public access shock AED**  **/**  **FR shock AED** | **FR arrival at scene**  **/**  **MICU arrival at scene** | **Non-shockable rhythm** | **Shockable rhythm** | **ROSC** | **Alive at day 30 or HD**  **/**  **CPC 1-2 at day 30 or HD** |
| --- | --- | --- | --- | --- | --- | --- | --- | --- | --- | --- | --- | --- |
| Negrello et al.  Martinique  Observational  2018-2019  N = 340 | 68  [54-78] | 64.0 | 74.7  /  20.0 | 69.1 | 91.8  /  8.2 | 51.2 | 0.6  /  7.9 | 20  [10-30]  /  27  [19-41] | 92.7 | 5.3 | 9.1 | 3.8  /  2.4 |
| Wiel et al.  French national ReAC  Case-control study  2011-2014  N = 8694  (33) | 64.5  [54-77] | 73.5 | 72.7  /  27.3 | 70.7 | 100  /  0 | 49.5 | - | - | 81.6 | 10.9 | 33.1 | 7.8  /  5.9 |
| Luc et al.  French national ReAC  Observational  2013-2014  N = 6918  (2) | 68  [53-82] | 63.2 | 75.0  /  14.0 | - | 87.8  /  12.2 | 49.0 | - | -  /  18  [12-25] | 86.3 | 5.9 | - | 4.9  /  - |
| Noel et al.  French national ReAC  Observational  2012-2018  N = 18,185  (24) | - | 70.1 | 76.5  /  - | 71.8 | 100  /  0 | - | 2.2  /  24.8 | -  /  20  [14-27] | 70.5 | 29.5 | 32.0 | 6.7  /  5.0 |
| Heidet et al.  French national ReAC  Observational  2014-2018  N = 3239  (25) | 71  [57-82] | 65.0 | 74.0  /  12.0 | 45.0 | 100  /  0 | - | 7.0  /  23.0 | 8  [5-12]  / 18  [13-23] | - | 7.0 | 21 | 5.0  /  4.0 |
| Javaudin et al.  French national ReAC & Paris-SDEC  Observational  2012-2020  N = 57,985  (46) | 69  [57-81] | 68.4 | 74.3  /  18.2 | - | 86.3  /  13.7 | 47.6 | - | - | - | 15.8 | - | 6.2  /  4.7 |

Abbreviations: AED: Automatized external defibrillator; CPC: Cerebral Performance Categories; CPR: CardioPulmonary Resuscitation; FR: First-responders; HD: hospital discharge; MICU: Mobile Intensive Care Unit; OHCA: Out-of-hospital cardiac arrest; ROSC: Return of spontaneous circulation. Quantitative variables are expressed as median with interquartile range, while qualitative variables are expressed as percentages.
